# Identifying and addressing disparities in the evaluation and treatment of children with growth hormone deficiency

**DOI:** 10.3389/fendo.2022.989404

**Published:** 2022-08-24

**Authors:** Kara Beliard, Vickie Wu, Julie Samuels, Terri H. Lipman, Robert Rapaport

**Affiliations:** ^1^ Division of Pediatric Endocrine and Diabetes, Mount Sinai Kravis Children’s Hospital, New York, NY, United States; ^2^ Department of Family and Community Health, University of Pennsylvania School of Nursing, Philadelphia, PA, United States

**Keywords:** growth hormone deficiency, pediatric short stature, gender disparities, racial disparities, healthcare disparities

## Abstract

Health disparities are a significant cause of concern globally and in the United States. Disparities have been additionally highlighted throughout the ongoing COVID-19 pandemic during which populations of color have been the most affected by the disease. Social determinants of health, race, ethnicity, and gender have all contributed to disparate outcomes and disparities spanning all age groups. Multiple socio-ecological factors contribute to disparities and different strategies have been proposed. The purpose of this paper is to provide an overview of disparities in pediatric treatment and outcomes, with a focus on children with endocrine disorders.

## Introduction

Health disparities are a significant cause of concern globally and in the United States. Disparities have been additionally highlighted throughout the ongoing COVID-19 pandemic during which populations of color have been the most affected by the disease (1, 2). Social determinants of health, race, ethnicity, and gender have all contributed to disparate outcomes and disparities spanning all age groups. Multiple socio-ecological factors contribute to disparities and different strategies have been proposed. The purpose of this paper is to provide an overview of disparities in pediatric treatment and outcomes, with a focus on children with endocrine disorders.

Health disparities are defined as differences in health outcomes that can be attributed to social, economic, or environmental disadvantages and frequently greatly affect health outcomes. For example, food insecurity in children leads to malnutrition, poor growth, weakened immune systems, and is a common cause of death globally (3). Most pediatric health disparity research has focused on the cumulative and synergistic impact of differences in socioeconomic status (SES), race, and ethnicity on the life-course trajectory and its outcomes in adulthood.

The Center for Disease Control (CDC) defines the social determinants of health as circumstances in which individuals are born, live, work, and age that impact health outcomes (3). This encompasses four categories of interacting factors: 1) socioeconomic circumstances, 2) psychosocial factors, 3) neighborhood environment, and 4) political, economic, and cultural drivers (4, 5).

Racial and ethnic disparities exist in health care and health outcomes in the U.S. across the socioeconomic spectrum. Minority groups have been found to have a higher incidence of complications and mortality associated with oncologic diseases, chronic diseases, and infant mortality (5, 6). Most recently, the COVID-19 pandemic has significantly and disproportionately impacted minority groups in the U.S. in prevalence, intensive care unit admissions, and deaths - exacerbating pre-existing disparities (7). Given that the 2020 U.S. Census Bureau reports non-White individuals comprise 42.2% of the population, the estimated number of individuals affected is staggering (8).

Prevalent gender disparities in healthcare are another major source of high impact inequities (9). For example, despite females having an increased survival post liver transplant compared to males, females have a lower probability of receiving a liver transplant (10). Compared to men with diabetes, women with diabetes have higher rates of coronary heart disease, stroke, depression, anxiety, and mortality (11).

## Health disparities in pediatric health care

Health care disparities have been extensively reported in the pediatric population. In pediatric emergency rooms children from minority groups had longer wait times and fewer analgesics prescribed in trauma cases (12). In Neonatal Intensive Care Units (NICU), compared to Black and Hispanic children, White children experienced higher rates of breastfeeding, more early intervention referrals, more kangaroo care, and had less risk of intraventricular hemorrhage, and lower rates of mortality and morbidity (13). Female infants compared to males had higher rate of post-cardiac surgery mortality (14). Even in pediatric clinical trials, representation of Black children has increased in the last decade, although still underrepresented, however disparities still exist in the enrollment of American Indian/Alaska Native, Asian, and Native Hawaiian/Pacific Islander children (15).

## Health disparity in pediatric endocrinology

Social, ethnic, and gender disparities are also noted in the field of Pediatric Endocrinology. Diabetes mellitus is one of the most common pediatric chronic diseases and significant advances have been made in managing this disease (16). Recent studies have demonstrated that Black children are significantly less likely to use continuous glucose monitors and insulin pumps (17–22). Data have shown that caregivers’ perception of cost and providers’ perception of family competence were essential factors when deciding the level of treatment intensity in patients with type 1 diabetes mellitus (T1DM) (23). When using health insurance as a proxy for SES, White children with public insurance were 1.4 to 1.7 times more likely than Black children with commercial insurance to be prescribed an insulin pump (17). Even when both parents completed high school and college, 68% of White children received diabetes technology compared to 34% of Black children (20).

Patients who receive less intensive treatment have poorer diabetes control. Studies on rates of complications associated with T1DM in minority groups reported that Hispanic and Black children have poorer metabolic control when compared to White children (24–27). White children were found to have lower hemoglobin A1c when compared to Black children, independent of insurance type (17). When assessing chronic complications of T1DM such as diabetic retinopathy, White children were more likely to obtain annual dilated eye examination screenings than Black children (28). Compared to White and Hispanic children, Black children have more hospital admissions for diabetic ketoacidosis and more hypoglycemia episodes (18). Black children represent 46% of all pediatric diabetes mellitus population yet comprise 77% of the diabetes-related deaths. In contrast, White children represent 26% of the pediatric diabetes mellitus population and comprise only 7% of related deaths (18).

Racial and gender disparities are also seen in the evaluation of childhood short stature (SS). SS is defined as height less than two standard deviations (-2 SD) below the mean for age and sex. Growth failure is defined as growth velocity <0 SD below the mean for age and sex. SS can be a normal variant of growth as seen in familial short stature and constitutional delay; however, it can also reflect pathological states (29–31). For a child to be evaluated for endocrine causes of SS, a referral is typically initiated by the primary care provider (PCP). Retrospective data analyses have reported a predominance of White males being referred for concerns of growth (22, 32). Caregivers’ attitudes and level of concern play an essential role in deciding when to refer a child to a subspecialty clinic and the degree of concern is not uniform amongst different families. When parents were questioned about the impact of SS on adult men and women, they reported that short men suffer in self-esteem and personal success; in contrast, short women were not believed to face these problems (33). Several studies have found that Black families had a higher threshold to consider SS an issue, believing that height is a minor problem when more important issues exist (34, 35). Explicit and implicit providers’ bias has been extensively reported and even the most well-meaning providers may have subconscious biases, known as implicit biases (7, 36). It is therefore crucial for health care providers to identify and address structural racism in health care team that may be perpetuating poor health outcomes in minority groups.

In the evaluation of children with SS associated with growth hormone deficiency (GHD), data from several growth hormone (GH) surveillance programs have highlighted gender, racial and ethnic disparities in diagnosis and treatment (**Table 1**). August et al. reported the demographics of children followed in the post-marketing surveillance of Somatrem; the population consisted of 87.8% White, 6% Black, and 1% Asian (40). Males comprised 71.6% of their population and females comprised 28.4%. They noted that Black children referred for SS were shorter and had lower peak GH levels during the growth hormone stimulation test (GHST) than their White counterparts. At diagnosis, females with idiopathic GHD were significantly shorter than males (-3.9 ± 1.3 SD versus -3.3 ± 1.4 SD, respectively). The KIGS worldwide registry (Pfizer International Growth Database) gathered data from 1987-2012 and included over 80,000 children with SS; of those evaluated and treated for GHD, 70% were White, 14.4% were East Asian, 1.1% were Black, and 2.4% were Hispanic, with a higher frequency of males in total (70.1% males versus 29.9% females) (41). Studies have reported that male White children and children from higher annual family income and parental education were more likely to be evaluated for SS, and be diagnosed and treated for GHD (33, 43). In one study, 91% of the families presenting for evaluation of their child’s SS were White and 5% were Black, yet the regional population is 72% White and 25% Black; in addition, 49% of the presenting families had annual incomes ≥ $50,000 however only 23% of families in the county had this level of income (33).

**Table 1:** Gender and Racial disparities in the diagnosis and treatment of Growth Hormone Deficiency.

Table 1A								
Paper		M:F % referral			M:F height z-score at time of referral			
Hawkes et al. (37)		61/39						
Kamoun et al. (38)		65/35			-1.8/-2.0			
Grimberg et al. (32)		65/35			-1.9/-2.4			
Tanaka et al. (39)		61.3/38.7			-2.47/-2.52			
**Table 1B**								
**Paper**					**W:B % referrals**			
Hawkes et al. (37)					79/11			
Kamoun et al. (38)					90/10			
**Table 1C**								
**Paper**	**M:F %, underwent GHST to diagnose IGHD**	**M:F %, diagnosed with IGHD**	**M:F %, received GH treatment for all GH indications**		**M:F %, received GH treatment for IGHD**	**M:F %, received GH treatment for ISS**	**M:F baseline height z-score, received GH treatment for IGHD**	
Grimberg et al. (34)			63/37					
August et al. (40)			70.5/29.5		71.6/28.4		-3.3/-3.9	
Ranke et al. (41)			67/33		70/30	71/29	-2.84/-3.22	
Kamoun et al. (38)	70/30				70/30		-2.2/-2.5	
Hughes et al. (42)			64/36		62/38	66/34	-2.092/-1.6	
Tanaka et al. (39)	45.7/49.8	61.4/38.6						
**Table 1D**								
**Paper**	**W:B %, underwent GHST to diagnose IGHD**	**W:B %, received GH treatment for all GH indications**		**W:B %, received GH treatment for IGHD**		**W:B %, received GH treatment for ISS**	**W:B, baseline height z-score, received GH treatment for IGHD**	**W:B, Maximal GH peak in ng/mL on GHST, received GH treatment for IGHD**
Grimberg et al. (34)		83/4		84/3		85/3	-2.6/-3.0	6.0/4.9
August et al. (40)		87.8/6.0					-3.4/-4.1	3.5/2.8
Hawkes et al. (37)	84/9			83/10			-2.3/-2.5	7.2/4.7

A- Percentage of females (F) and males (M) referred to Pediatric Endocrinology for short stature assessment with their corresponding initial height z-scores. B- Percentage of White (W) and Black (B) children referred to Pediatric Endocrinology for short stature assessment. C- Percentage of females (F) and males (M) assessed and treated for Growth Hormone Deficiency with their corresponding initial height z-scores. D- Percentage of White (W) and Black (B) children assessed and treated for Growth Hormone Deficiency with their corresponding initial height z-scores and Growth Hormone peak during the Growth Hormone Stimulation Test (GHST).

- IGHD, Idiopathic Growth Hormone Deficiency.

- GH, Growth Hormone.

- GHST, Growth Hormone Stimulation Test.

- ISS, Idiopathic Short Stature.

Compared to females, males are more likely to be screened for GHD by PCPs (44). White male children are referred more often for SS evaluation when compared to minority groups and females (37). As part of the evaluation for SS, Pediatric Endocrinologist may perform a GHST. This test is performed after an overnight fast and involves administering provocative agents (clonidine, arginine, glucagon, insulin) and obtaining serial GH concentrations. Disparities are seen with GHST, with more White males proceeding with the test than minority groups and females (37). These studies also highlight the high proportion of females not being assessed for SS; this is distressing as SS can be the only physical examination finding in patients with Turner Syndrome (38, 39, 42, 44, 45). Grimberg et al. reported a higher rate of pathological/organic causes of SS in females in their evaluation, even when excluding Turner Syndrome as a cause (32).

## Proposed interventions

Over the years, multiple authors have highlighted these health disparities and proposed different interventions to reduce and eventually eliminate disparities in healthcare. The underrepresentation of minority children and females being evaluated and treated for short stature due to GHD is striking and demands attention.

**Figure 1:**
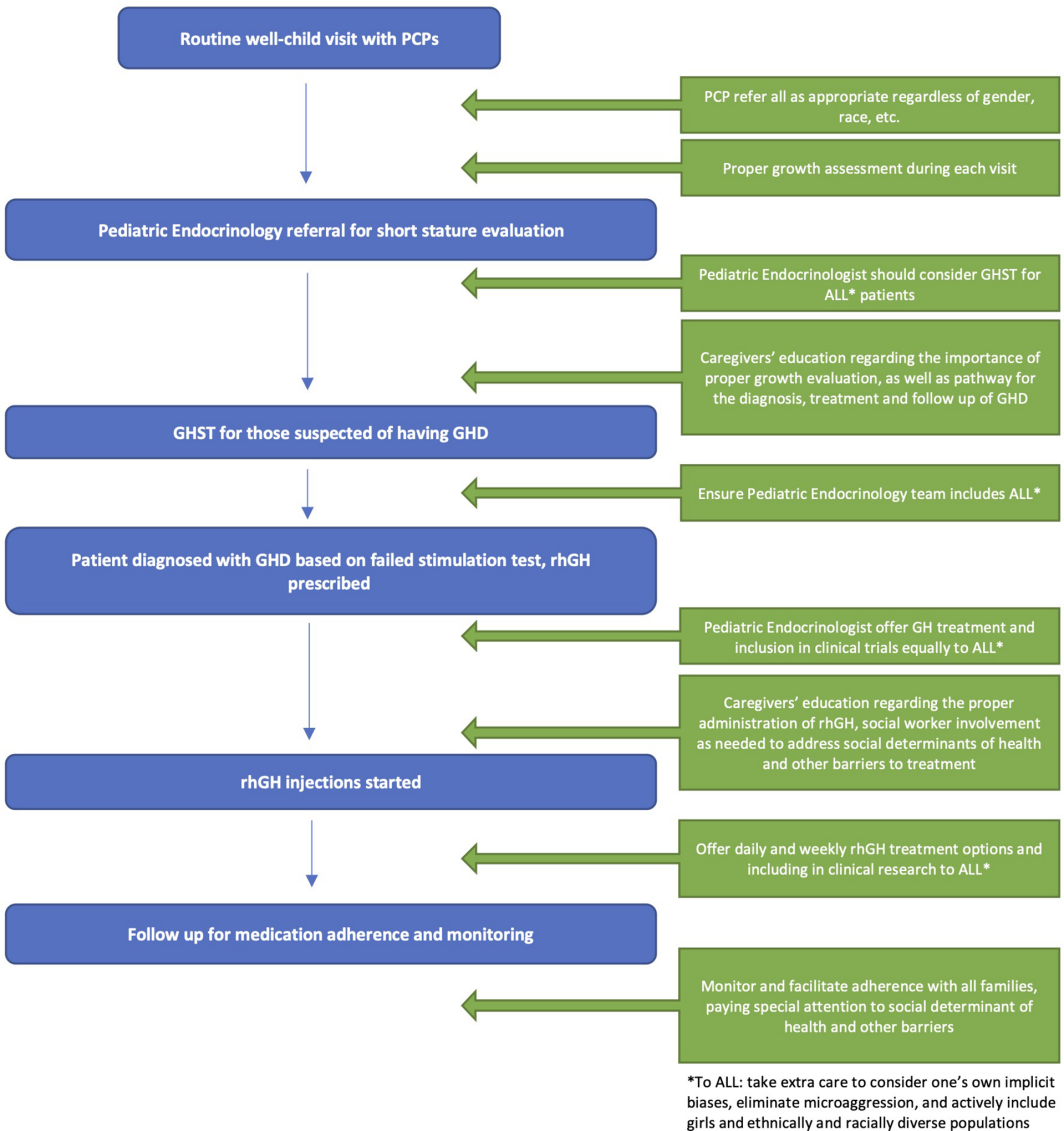
Proposed Interventions.

We propose the following interventions to address the aforementioned issues, focusing on pediatric GHD **Figure 1** .

• EDUCATE FAMILIES REGARDING THE IMPORTANCE OF HEALTH MAINTENANCE VISITS FOR CHILDREN
o The recommended schedule for health maintenance visits is at the first week of life, 1 month, 2 months, 4 months, 6 months, 9 months, 12 months, 15 months, 18 months, 24 months, 30 months, 3 years, and annually thereafter.
o Families should be educated about the importance of scheduling and attending the health maintenance visits.
• DEMAND KNOWLEDGE OF GROWTH PARAMETERS LONGITUDINALLY
o At every visit with the PCP, families should receive information about their child’s growth parameters – both the absolute measurements and the percentiles for age and sex.
• WHEN GROWTH PATTERNS DEVIATE, STRESS THE IMPORTANCE OF EVALUATION
o When growth patterns deviate, evaluation is crucial to distinguish between normal variants of growth such as constitutional delay of growth, conditions unrelated to hormonal causes such as poor nutrition or celiac disease, and endocrinopathies such as GHD.
• EDUCATE COMMUNITY PROVIDERS REGARDING APPROPRIATE EVALUATION AND REFERRAL OF CHILDREN WITH CONCERNS FOR GHD
o The decision to refer a child for an evaluation for endocrine causes of SS should be standardized. The American Academy of Pediatrics (AAP) recommends monitoring growth parameters (height and weight) at each health maintenance visit as an effective general health and well-being assessment method.
o Correct height measurement techniques must be implemented to prevent growth failure from being unrecognized or misdiagnosed (46). All health care team members responsible for obtaining height measurements should receive training on proper technique and equipment.
o When children exhibit abnormal growth patterns, PCPs must perform a proper diagnostic evaluation that may include referral to a Pediatric Endocrinologist.
o The PCP’s decision to pursue evaluation of SS should be irrespective of a child’s gender or race.
• EDUCATE PARENTS AND CAREGIVERS REGARDING GHD, ITS COMPLICATIONS, AND TREATMENT OPTIONS
o Parental education should focus on complications associated with childhood GHD, including the impact on bone health, lipid profile, psychosocial state, and well-being.
o The indications for recommending GHST should be clearly explained to families and all questions should be addressed to limit concerns.
o Parents may have misconceptions regarding the safety of recombinant human GH. The medication has been historically associated with Creutzfeldt-Jakob disease linked to contaminated human GH obtained from the cadaveric human pituitary gland. Cases of Creutzfeldt-Jakob disease associated with GH treatment are no longer a concern since the transition to recombinant human GH (rhGH) in 1985 (47).
o Families may be hesitant to start the medication for fear of daily injections. However, weekly rhGH is available and FDA approved to be used in the pediatric population.
• EDUCATE PEDIATRIC ENDOCRINOLOGISTS WITH LATEST GHD RESEARCH AND TREATMENT OPTIONS
o Pediatric Endocrinologists should be knowledgeable about the latest GHD research and treatment options.
o Pediatric Endocrinologists should also be knowledgeable in how to administer rhGH to appropriately address patients’ and families’ questions or troubleshoot issues with administration.
• ADDRESSING THE DISPARITIES OBSERVED IN GHST WHEN CLINICALLY INDICATED
o GHST should be offered to all children in whom GHD is suspected, regardless of race, ethnicity, or gender.
o The decision to undergo GHST should not be influenced by parental perceptions of height outcomes in males versus females. Providers should stress the indications for GHST and the importance of treatment of GHD not just for height attainment.
o Provider bias, if present, should be recognized and should not interfere with the recommendation of GHST if clinically warranted.
o Pediatric Endocrinologists should strive to identify their own potential biases and barriers to offering and providing equal care to males and females, regardless of race, ethnicity, and SES.
• EVALUATION WITH MRI
o After the clinical and biochemical diagnosis of GHD is made, obtaining magnetic resonance imaging (MRI) is recommended to evaluate the hypothalamic pituitary region. It can identify pituitary abnormalities such as anterior pituitary hypoplasia, posterior pituitary ectopia, and pituitary stalk agenesis. MRI can also exclude the presence of a pituitary tumor.
o The role of MRI should be clearly explained to the family and recommended to all patients if indicated.
• GH TREATMENT INITIATION
o GH therapy should be offered equally to all patients who meet the diagnostic criteria for GHD.
• SUPPORT DURING GH TREATMENT
o Promoting inclusivity amongst families with children with GHD is encouraged. Connecting families with a new diagnosis of GHD with families who are actively receiving or who have completed treatment with rhGH to establish/promote more community support may ease the caregivers’ concerns.
o If there are financial or social barriers to initiating GH treatment, the Pediatric Endocrinology office should have effective intervention options in place, such as social workers available to identify resources to help mitigate these concerns.
• MONITORING OUTCOMES DURING AND AFTER GH TREATMENT
o Follow-up visits in the office are typically every three months for close monitoring of height, side effects, and dose adjustments. Efforts should be made to support patients and caregivers for them to be able to attend these visits in the form of appointment availability and flexibility.
o When GH treatment is discontinued, patients and families should continue to follow up at the Pediatric Endocrinology office.
• PROMOTE DIVERSITY OF THE HEALTH CARE TEAM
o Efforts must be made to increase diversity in the health care team – in ethnicity, race, and gender.

## Discussion

Health care disparities have been a subject of discussion for decades however we have yet to find the best way to address this ongoing concern. Culturally competent, equitable care that is sensitive to patients’ needs should be the priority. The patients’ health literacy and cultural beliefs should be considered when discussing these matters with the families (7). Well-meaning providers may have implicit biases that may impact their decision when referring children for SS evaluation, proceeding with GHST, and ensuring appropriate treatment and follow-up for different patient populations. Providers’ unintentional more positive attitudes towards White patients and negative attitudes towards ethnically and racially diverse groups can impact patient care (48, 49). Many have postulated that racial diversity in the medical field is an essential step in addressing racial disparities. Healthcare providers need to examine their own practices to ensure elimination of unconscious or overt biases that can perpetuate microaggression in the patient-provider relationship. It is imperative that providers address structural racism and its role in perpetuating health disparities (22).

Health care disparities have significantly and disproportionally impacted minority populations. This is seen in various areas of health care, but the conclusion remains unchanged, with underrepresented groups having worse outcomes. Different solutions have been postulated – this includes educating caregivers, improving the social determinants of health of patients, educating, and diversifying healthcare providers, and addressing and alleviating implicit and explicit bias in healthcare providers. In the field of pediatric endocrinology, we propose steps to advance equity in the evaluation, diagnosis, and treatment of children for GHD. A multidisciplinary approach is needed to minimize implicit or explicit bias, to encourage collaboration between members participating in patient care, and to support families through the treatment of GHD.

## Author contributions

KB and VW wrote the manuscript. JS contributed to the editing as well as the research and addition of reference material. TL and RR helped develop the initial outline and significantly contributed to the content and editing of the manuscript. All authors contributed to the article and approved the submitted version.

## Conflict of interest

The authors declare that the research was conducted in the absence of any commercial or financial relationships that could be construed as a potential conflict of interest.

## Publisher’s note

All claims expressed in this article are solelyB those of the authors and do not necessarily represent those of their affiliated organizations, or those of the publisher, the editors and the reviewers. Any product that may be evaluated in this article, or claim that may be made by its manufacturer, is not guaranteed or endorsed by the publisher.
